# Which healthy lifestyle activities are associated with better mental health outcomes?

**DOI:** 10.1192/j.eurpsy.2026.11853

**Published:** 2026-07-31

**Authors:** S. K. K. Lam, M. H. Lo, M. E. S. Reyes, E. S. Jaya, F. Mukhtar, A. E. Z. Lian, G. Derin, P. D. Bengwasan, K. Uludag, A. K. C. Chau, H. W. Fung

**Affiliations:** 1 School of Nursing and Health Sciences, Hong Kong Metropolitan University; 2 Department of Social Work and Social Administration, The University of Hong Kong, Hong Kong, Hong Kong; 3 Department of Psychology, College of Science, University of Santo Tomas, Manila, Philippines; 4 Faculty of Psychology, Universitas Indonesia, Depok, Indonesia; 5 Department of Psychiatry, Universiti Putra Malaysia, Selangor; 6 Faculty of Social Sciences, Raffles University, Johor, Malaysia; 7 Psychotraumatology and Psychohistory Research Unit, Istanbul University-Cerrahpaşa, İstanbul, Türkiye; 8 Department of Psychology, De La Salle University, Manila, Philippines; 9 School of Medicine, Shanghai Jiao Tong University, Shanghai, China; 10 Department of Psychiatry, The University of Hong Kong; 11 School of Nursing, The Hong Kong Polytechnic University, Hong Kong, Hong Kong

## Abstract

**Introduction:**

Healthy lifestyle is a protective factor against mental health problems. Yet, less is known about which specific lifestyle activities would be associated with diverse mental health outcomes.

**Objectives:**

This study examined whether different healthy lifestyle activities would predict psychiatric symptoms.

**Methods:**

We analyzed longitudinal data from 293 female mental health service users from diverse regions (36.9% from Western countries). They provided data two times, approximately seven months apart. They answered questions regarding specific lifestyle activities (“often spend time looking at trees, flowers and other natural elements”, “have a pet”, “often do physical activity or exercise”, and “often consume ≥4.5 cups/day of fruits and vegetables, ≥2 servings/week of fish”) and completed the Patient Health Questionnaire, the International Trauma Questionnaire, the Multiscale Dissociation Inventory, and the Somatic Symptom Scale-8. For each analysis, we controlled for baseline scores, age, education level and regions in the hierarchical linear regression analysis and examined whether specific lifestyle activities would predict the scores at Time 2.

**Results:**

Baseline consumption of fruits and vegetables negatively predicted subsequent levels of depressive symptoms (β = -.139, p = .002), complex PTSD symptoms (β = -.134, p = .003), and dissociative symptoms (β = -.130, p = .002). These symptoms were not predicted by other measured lifestyle activities. Somatic symptoms were not predicted by any of the measured lifestyle activities.

**Conclusions:**

Consuming fruits and vegetables predicted fewer symptoms of depression, complex PTSD, and dissociation. Yet, it remains unclear whether the protective effect stems from the foods themselves or related factors, such as healthy eating habits. Further experimental research is needed to clarify these associations.

**Disclosure of Interest:**

None Declared

